# The Effect of Early vs Delayed Initiation of Adalimumab on Remission Rates in Patients With Crohn’s Disease With Poor Prognostic Factors: The MODIFY Study

**DOI:** 10.1093/crocol/otab064

**Published:** 2021-09-04

**Authors:** Gerassimos J Mantzaris, Christos Zeglinas, Angeliki Theodoropoulou, Ioannis Koutroubakis, Eleni Orfanoudaki, Konstantinos Katsanos, Dimitrios Christodoulou, Georgios Michalopoulos, Maria Tzouvala, Dimitrios Moschovis, Spyridon Michopoulos, Evanthia Zampeli, Konstantinos Soufleris, Anastasios Ilias, Christina Chatzievangelinou, Antonios Kyriakakis, Konstantia Antachopoulou, Konstantinos Karmiris

**Affiliations:** 1 Department of Gastroenterology, General Hospital of Athens “Evaggelismos”, Athens, Greece; 2 Medical Department, AbbVie Pharmaceuticals S.A., Athens, Greece; 3 Department of Gastroenterology, General Hospital of Heraklion “Venizeleio-Pananeio”, Heraklion, Crete, Greece; 4 Department of Gastroenterology, University Hospital of Heraklion, Crete, Greece; 5 Department of Gastroenterology, Pathology Unit, University General Hospital of Ioannina, Ioannina, Greece; 6 Department of Gastroenterology, General Hospital of Piraeus “Tzaneio”, Piraeus, Greece; 7 Department of Gastroenterology, General Hospital of Nikaia & Piraeus “Agios Panteleimon”-General Hospital Dytikis Attikis “Agia Varvara”, Nikaia, Greece; 8 Department of Gastroenterology, Pathology Unit, General Hospital of Athens “Alexandra”, Athens, Greece; 9 Department of Gastroenterology-Oncology, Pathology Unit, Anticancer Hospital of Thessaloniki “Theageneio”, Thessaloniki, Greece; 10 Department of Gastroenterology, Pathology Unit, General Hospital of Thessaloniki “G. Papanikolaou”, Thessaloniki, Greece

**Keywords:** adalimumab, early Crohn’s disease, prognostic factors

## Abstract

**Background:**

Data on the effectiveness of anti-tumor necrosis factor medications in patients with Crohn’s disease (CD) with poor prognostic factors (PPFs) are scarce. This study aimed to generate real-world evidence on the effect of early (≤24 months after diagnosis) vs delayed (>24 months) initiation of adalimumab (ADL) on the 26-week remission rate (Harvey–Bradshaw Index ≤4) in these patients.

**Methods:**

This multicentre, retrospective, chart review study performed in 10 Greek hospitals enrolled adult patients with moderate to severe CD (Harvey–Bradshaw Index ≥8) with ≥3 PPFs who were initiated on ADL ≥12 months before enrollment. A sample size of 164 patients (early:delayed cohort allocation ratio, 30:70) was required to address the primary endpoint.

**Results:**

Eligible patients (*n* = 171) were consecutively enrolled. In the early vs delayed cohorts, the 26-week remission rates (off-steroids) using the last-observation-carried-forward imputation method were 60.7% (37/61) vs 47.2% (50/106), respectively (Δ = 13.5%, *P* = .044). The respective remission rates were 61.2% vs 42.4% among anti-tumor necrosis factor-naive patients (*P* = .023) and 58.3% vs 53.2% among anti-tumor necrosis factor-experienced patients (*P* = .374). The 52-week remission rates using as-observed data were 78.8% and 60.3%, and the intestinal resection rates were 6.5% and 11.9% in the early vs delayed ADL cohorts, respectively.

**Conclusions:**

Patients with CD with PPFs who received early vs delayed treatment with ADL achieved higher clinical response and remission rates. This effect was more pronounced in those patients who were bio-naive and steroid-dependent/refractory with concurrent extraintestinal manifestations than those who were not.

## Graphical Abstract

**Figure:**
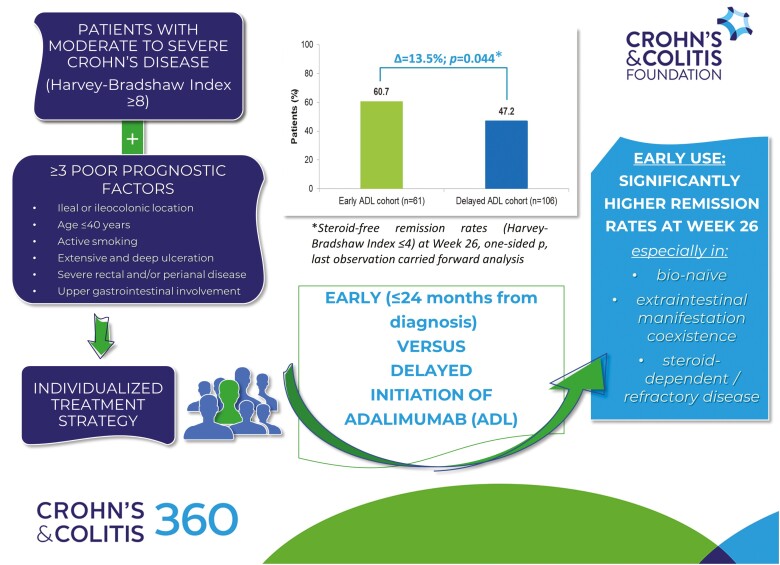

## Introduction

Crohn’s disease (CD) is a chronic disease with dynamic progression towards complicated phenotypes,^1^ resulting in disabling symptoms.^2^ Identification of prognostic factors associated with certain long-term poor outcomes has been the focus of several epidemiologic studies.^3–7^ The identification and validation of prognostic factors is a major objective in clinical research, not only as a means to predict the natural history of CD, but also to apply highly individualized therapeutic strategies that would yield optimized disease outcomes by avoiding both over- and under-treatment.^8, 9^

However, to halt the progressive bowel damage in CD beyond the identification of poor prognostic factors (PPFs) early treatment with disease-modifying drugs, such as anti-tumor necrosis factor (TNF) agents, is also required.^10, 11^ Early CD is defined as an active, inflammatory disease with objective signs of disease activity but no bowel damage and a disease duration of <2 years.^12^ This definition has been based on the paradigm of rheumatoid arthritis,^13, 14^ taking into consideration the fact that anti-TNFs [adalimumab (ADL), infliximab, certolizumab pegol] are more effective in patients with disease duration of <2 years.

Even though it is well recognized that identifying PPFs would allow individualized, early anti-TNF intervention, data on the effectiveness of anti-TNFs in patients with CD with poor prognosis are scarce. This study aimed to generate real-world evidence on the effect of early vs delayed initiation of ADL in patients with PPFs. Further, the potential association between early vs delayed initiation of ADL in the CD course and prevention of poor long-term outcomes was examined.

## Materials and Methods

### Study Design and Setting

This single-country, multicentre, retrospective, chart review study was performed by gastroenterologists specialized in inflammatory bowel disease (IBD) in 10 IBD centers of public hospitals in Greece. Sites were selected through a documented and structured feasibility process that accounted for physicians’ qualifications, previous participation and experience in similar studies, number of potentially eligible patients, data availability, and staff resourcing. To represent variations in current real-world patterns of care, research sites were recruited from various geographic regions in Greece, taking into consideration the regional setting and type of healthcare site/institution (~31% of the patients recruited from academic and the remaining 69% from nonacademic sites) as well as the hospital activity.

The study involved secondary data collection from medical charts of patients with CD treated with ADL by means of an electronic web-based data capture system. Medical charts were reviewed and assessed through a process of consecutive sampling that followed reverse chronologic order based on the date of ADL treatment onset. Beginning with the most recently ADL-initiated patients (ie, 12 months before selection process onset) and then moving retrospectively, patients were assessed for study-specific eligibility criteria. All eligible patients who were alive at the time of chart abstraction initiation were contacted by the site staff to provide consent on the use of their medical charts. If the eligible patient could not be reached during the initial telephone contact, site staff attempted telephone contact 3 more times before considering the eligible patient to be not interested in study participation. Eligible patients who attended the participating clinical sites within the context of a routine clinical visit during the selection process period were also considered for inclusion in the study.

The study look-back period for data collection comprised (i) the pre-ADL initiation period, spanning from the date of CD diagnosis to the day before ADL treatment initiation and (ii) the period after ADL initiation, which began on the date of ADL treatment initiation and ended at loss to follow-up, chart abstraction initiation (for patients for whom ADL was ongoing at enrollment), a switch to another treatment, or at 3 months after ADL discontinuation, whichever came first.

### Study Population

The study population involved adult male and female consenting patients with moderately to severely active CD [Harvey–Bradshaw Index (HBI) score ≥8],^15^ who were naive or experienced to biologic therapy at initiation of ADL and who had ≥3 PPFs at CD diagnosis. The rationale for requiring the presence of ≥3 PPFs was based on the European Crohn’s and Colitis Organisation guidelines advising early treatment with thiopurines and/or biologics when two or more predictors are present.^16^ PPFs included ileal or ileocolonic location, age ≤40 years, active smoking, extensive and deep ulceration, severe (as per physician judgment) rectal and/or perianal disease, and upper gastrointestinal involvement. Patients had initiated treatment with ADL, as per the approved label, ≥12 months before enrollment in the study and after it was launched in the Greek market (June 2007). Patients should also have had sufficient available medical records for data abstraction to meet the objectives of the study. Patients with a history of CD-related intestinal resection before initiation of ADL, those who had participated in any clinical trial with any investigational product/intervention during treatment with ADL, as well as female patients who had become pregnant during ADL treatment were excluded.

Patients were stratified into 2 cohorts, according to the length of CD duration at ADL initiation. Those with CD duration of ≤24 months before initiation of ADL were stratified in the early cohort; those with disease duration of >24 months were placed in the delayed cohort. The study population was recruited through the implementation of a competitive recruitment strategy, until the planned sample size was reached, at a targeted allocation ratio of 30:70 between the early and delayed cohorts.

### Study Outcomes

The primary objective of the study was to examine the effect of early vs delayed ADL initiation on the 26 ± 4-week post-baseline clinical remission rate by estimating the difference in the remission (HBI score ≤4) rates (off-steroids) between the 2 cohorts. Baseline was defined as the date of ADL treatment initiation (first injection) or the closest time point before that date. Secondary study outcomes included (i) the difference between the early and delayed cohorts in the 26 ± 4-week off-steroids clinical response rate, defined as a ≥3-point decrease in the HBI score; (ii) the relative risk of intestinal resection (excluding surgeries for perianal disease) among patients in the early and delayed cohorts; (iii) the relative risk of CD progression from an inflammatory to a stricturing and/or penetrating phenotype among patients in the 2 cohorts; and (iv) the difference between the 2 cohorts in the proportion of patients remaining on treatment at week 52 ± 4 (drug survival rate).

### Definitions Relating to PPF and Baseline Characteristics of Interest

The following are PPFs of interest and their definitions: active smoking, defined as smoking >7 cigarettes per week for ≥6 months immediately before CD diagnosis; extensive and deep ulceration, defined as large coalescent and deep ulcerations covering more than 10% of the mucosal area of ≥1 segment of the rectocolon; and upper gastrointestinal involvement, defined as esophageal, gastric, duodenal or jejunal inflammation due to CD.

The following are the baseline factors of interest: (a) the presence of extensive intestinal mucosal inflammation, defined as intestinal CD affecting >100 cm regardless of location of the inflammation; (b) being steroid-dependent, defined as either being unable to reduce steroids below the equivalent of prednisolone 10 mg/day (or budesonide below 3 mg/day) within 3 months of starting steroids without recurrent active disease or having had a relapse within 3 months of stopping steroids; and (c) being steroid-refractory, defined as having active disease despite treatment with prednisolone up to 1 mg/kg/day for a period of 4 weeks.

### Sample Size Estimation

The sample size calculation was based on the study’s primary endpoint. To detect a difference of 20.0% between the early vs delayed treatment cohorts with 80% power and an assumed 40.0% rate in the early treatment group,^17, 18^ a sample size of 164 was required, assuming a study size allocation ratio of 30:70 (early:delayed) and a 10% non-evaluable rate, using a 1-sided chi-square test at a 0.05 significance level. The sample size was calculated with SAS statistical analysis software version 9.4 using the PROC POWER TwoSampleFreq statement with the TEST = PChi and NFRACTIONAL options (SAS Institute, Cary, NC, USA).

### Statistical Analysis

Statistical analysis was performed using SAS software version 9.4. The normality of distribution was examined using the Shapiro–Wilk test. Data are presented as mean (SD) when normally distributed and as median [interquartile range (IQR)] when not. Regarding binomial proportions, 95% Wald CIs have been estimated. The difference between the 2 cohorts in the HBI remission and clinical response rates were estimated, and the statistical significance of the difference was examined with a Wald-based *z*-test statistic (1-sided test; a = 5%). Analyses of 26-week HBI remission rates and clinical response rates have been performed using the last-observation-carried-forward (LOCF), nonresponder imputation (NRI) methods, and as-observed data. To evaluate potential differences in the primary endpoint among selected subgroups, the difference in the HBI ≤4 attainment rate (LOCF imputation method) between the 2 study cohorts was examined and is presented separately in the subgroups per prior anti-TNF exposure (anti-TNF-naive, anti-TNF-experienced), steroid dependence/refractoriness, and the presence of extraintestinal manifestations at baseline. Differences between the values of continuous variables in 2 time periods were evaluated using a parametric statistical test (ie, paired *t*-test) for normally distributed data or its nonparametric analog (ie, Wilcoxon signed-rank test). Kaplan–Meier analysis was performed for the estimation of the median time to intestinal resection and progression to a stricturing/penetrating phenotype. Differences in the survival distributions between the 2 cohorts were examined with the log-rank test. The effect of study cohort (early vs delayed) on the rates of intestinal resection/progression to a stricturing/penetrating phenotype was evaluated using hazard ratios (HRs) and 95% CIs estimated from Cox proportional hazards models.

Finally, longitudinal analysis of C-reactive protein (CRP) over time was performed to assess the effect of early vs delayed ADL onset on CRP measurements obtained between ADL treatment onset and the end of the observation period. Repeated measures of CRP were used as the dependent variable and study cohort (early, delayed) time elapsed from ADL treatment onset to assessment (in weeks) and their interaction as independent variables. The Akaike information criterion (AIC) was used to compare linear mixed-effects/marginal models with different covariance structures; the model with the smallest AIC value was chosen. The interaction between cohort and time was not statistically significant, so inference was based on a model (fitted using the same covariance structure) including only the main effects. Unless specifically stated, statistical tests were 2-sided and were performed at a 0.05 significance level.

## Ethical Considerations

The study was designed and conducted in compliance with all applicable local laws and regulations, the Good Pharmacoepidemiology Practices of the International Society for Pharmacoepidemiology and the ethical principles laid down in the Declaration of Helsinki. The study was approved by the institutional review boards of the participating hospital sites. All patients provided written informed consent.

## Results

### Patient Disposition

**Figure 1.**
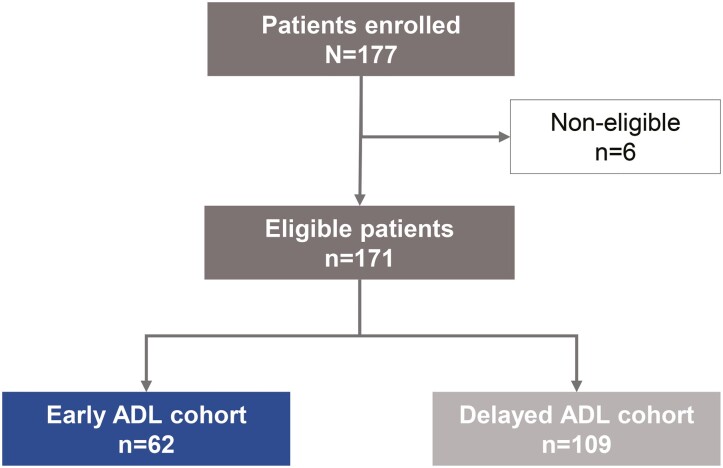
Patient disposition. ADL, adalimumab.

Eligible patients (*n* = 171; all White) who initiated ADL between July 1, 2007 and February 8, 2017 were consecutively enrolled in the study between October 20, 2017 and April 1, 2018; 62 (36.3%) were allocated to the early cohort and 109 (63.7%) to the delayed cohort (Figure 1).

The overall median (IQR) study look-back period between CD diagnosis and the end of the study observation period was 45.5 (28.8–75.5) and 148.2 (96.0–203.5) months for the early and delayed cohorts, respectively. The median (IQR) observation period after ADL treatment initiation was 37.7 (19.9–61.9) and 38.5 (22.4–70.9) months, respectively.

### Poor Prognostic Factors at CD Diagnosis

**Figure 2.**
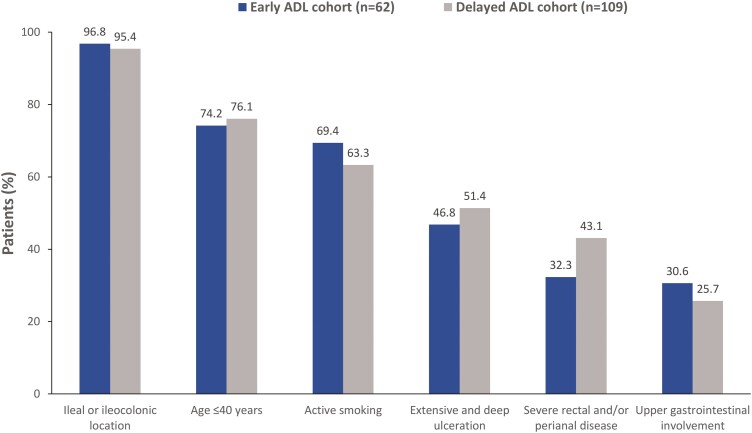
Poor prognostic factors at CD diagnosis in the early and delayed ADL cohorts. ADL, adalimumab; CD, Crohn’s disease.

A median (IQR) of 3 (3–4) of the protocol-specified PPFs were present in each cohort at diagnosis (Figure 2). In particular, 58.1% of the patients in the early cohort had 3, 33.9% had 4, and 8.1% had 5 PPFs. In the delayed cohort, 56.0% had 3, 35.8% had 4, and 8.3% had 5 or 6 PPFs. The 3 most common PPF in both cohorts were ileal or ileocolonic disease location, age ≤40 years, and active smoking (Figure 2).

### Baseline Characteristics

**Figure 3.**
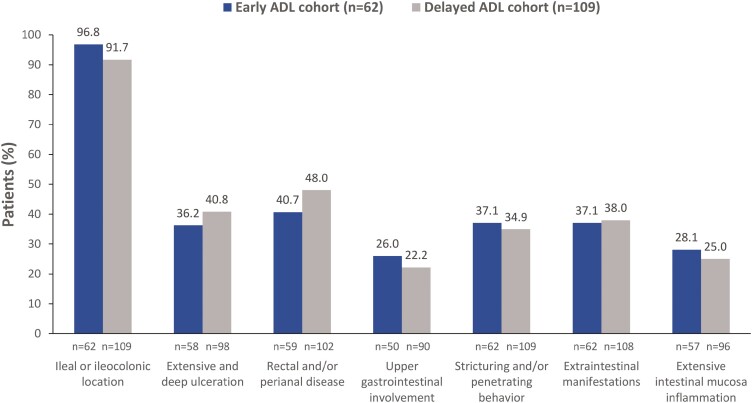
Baseline disease characteristics in the early and delayed ADL cohorts. ADL, adalimumab.

Characteristics of the early and delayed cohorts at baseline (ADL initiation) are presented in Table 1. At baseline, a median (IQR) of 5.3 (3.0–13.8) and 95.8 (48.0–140.7) months had elapsed from CD diagnosis to ADL initiation for the early and delayed cohorts, respectively. The 2 cohorts were not statistically different regarding essential disease characteristics (chi-square test, *P* > .05; Figure 3). Extraintestinal manifestations were present in 37.1% of the patients in the early and 38.0% of the patients with available data in the delayed ADL cohort. The most common extraintestinal manifestations (ie, in at least 3 patients) in the early ADL cohort were axial arthritis [in 19.4% (12/62)], peripheral arthritis [in 16.1% (10/62)], and oral aphthous ulcers [in 4.8% (3/62)]. Similarly, in the delayed ADL cohort, the most common extraintestinal manifestations were peripheral arthritis [in 27.8% (30/108)], axial arthritis [in 16.7% (18/108)], oral aphthous ulcers [in 3.7% (4/108)], and nephrolithiasis [in 2.8% (3/108)].

**Table 1.** Patient characteristics at baseline

	Early ADL cohort		Delayed ADL cohort	
	n	Value	N	Value
Age (years), median (Q25, Q75)	62	29.1 (23.6, 39.3)	109	37.8 (29.4, 47.0)
Males, *n* (%)	62	36 (58.1)	109	49 (45.0)
Caucasian, *n* (%)	62	62 (100.0)	109	109 (100.0)
BMI, (kg/m^2^), median (Q25, Q75)	44	23.6 (21.4–25.3)	64	23.3 (19.8–26.4)
At least one comorbidity, *n* (%)	62	9 (14.5)	109	14 (12.8)
Steroid dependency, *n* (%)	54	15 (27.8)	93	21 (22.6)

Abbreviations: ADL, adalimumab; BMI, body mass index; Q25, 25th percentile; Q75, 75th percentile.

At least one clinically significant (according to physician’s judgment) past or ongoing disease or surgery (other than those related to CD) was reported for 19.4% and 18.3% of the patients in the early and delayed cohorts, respectively. Before ADL initiation, 2 patients (3.2%) in the early cohort and 3 (2.8%) in the delayed cohort had undergone CD-related surgeries (seton placement, duodenal ulcer repair, and abdominal operation) other than intestinal resection.

### CD Treatment Characteristics

In the early and delayed cohorts, 79.0% and 100% of patients, respectively, had received ≥1 therapeutic regimen before ADL initiation (Table 2). In total, 19.4% of the patients in the early ADL cohort and 45.9% of those in the delayed ADL cohort had received prior biologic therapy, which comprised of anti-TNF agents in all cases. The main reasons for discontinuation of prior anti-TNF therapy in the early ADL cohort were adverse event/intolerance (in 66.7% of the cases) and loss of response (in 33.3%). In the delayed ADL cohort, the main reasons for discontinuation of prior anti-TNF therapy were loss of response (in 44.2% of the cases), adverse event/intolerance (in 34.6%), and inadequate response (in 9.6%).

**Table 2.** Prior and concomitant treatment characteristics

	ADL cohort	
Treatment, n (%)	Early (n = 62)	Delayed (n = 109)
Prior treatment	49 (79.0)	109 (100)
Prior exposure to biologic treatment (anti-TNF)	12 (19.4)	50 (45.9)
Prior exposure to non-biologic treatment	48 (77.4)	108 (99.1)
Corticosteroids	38 (61.3)	86 (78.9)
Azathioprine/6-mercaptopurine	22 (35.5)	68 (62.4)
Methotrexate	3 (4.8)	10 (9.2)
Concomitant medications	23 (37.1)	57 (52.3)
Immunosuppressive agents	17 (27.4)	37 (33.9)
Azathioprine	12 (19.4)	25 (22.9)
Methotrexate	5 (8.1)	10 (9.2)
6-Mercaptopurine	1 (1.6)	3 (2.8)
Corticosteroids	4 (6.5)	18 (16.5)
5-Aminosalicylates	4 (6.5)	8 (7.3)
Antibiotics	–	5 (4.6)

Abbreviations: ADL, adalimumab; TNF, tumor necrosis factor.

During a median period of ADL treatment of 37.7 months (early cohort) and 38.4 months (delayed cohort), 3.4% (2/58) and 6.4% (7/109) of the evaluable patients, respectively, had undergone temporary treatment interruptions, and 43.1% (25/58) and 41.3% (45/109) had undergone dose escalations.

The 52-week ADL survival rate exceeded 90% in both cohorts—91.9% (57/62 [95% CI, 85.2–98.7]) in the early cohort and 95.4% (104/109 [95% CI, 91.5–99.3]) in the delayed cohort—with no significant differences between the 2 cohorts (Δ = −3.5% [95% CI, −11.3, 4.4]; *P* = .384).

### Remission and Clinical Response Rates at 26-Week Post-Baseline

**Figure 4.**
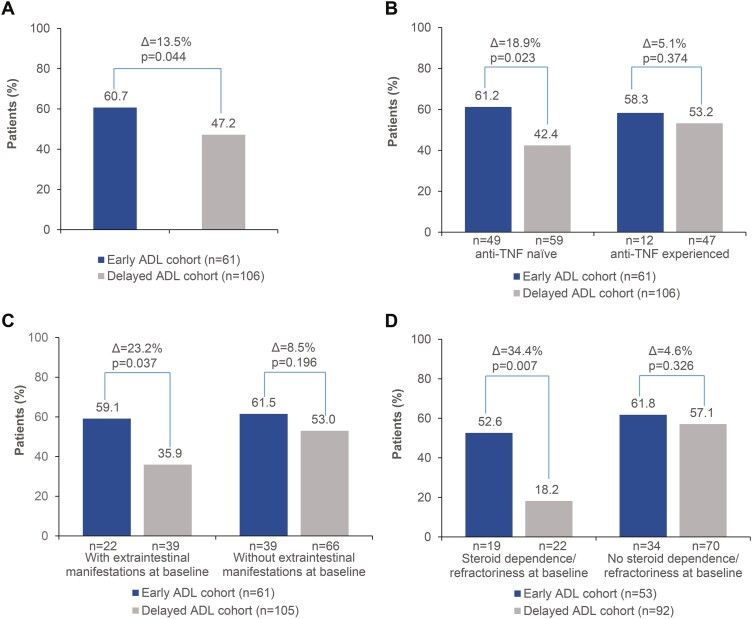
HBI off-steroids remission rates at week 26 after ADL initiation in the overall population and subpopulations of interest. (A) Overall population, (B) subgroups per prior exposure to anti-TNFs, (C) subgroups per the presence of extraintestinal manifestations at baseline, (D) subgroups per steroid dependency/refractoriness at baseline. One-sided chi-square *P* values and differences between the 2 groups (Δ) are shown above the bars. All analyses presented were performed using the last-observation-carried-forward imputation method. ADL, adalimumab; HBI, Harvey–Bradshaw Index; TNF, tumor necrosis factor.

The 26-week off-steroids remission rates (as defined by an HBI score of ≤4) using the LOCF imputation method were 60.7% (37/61 [95% CI, 48.4–72.9]) and 47.2% (50/106 [95% CI, 37.7–56.7]) in the early and delayed cohorts, respectively (1-sided *P* = .044; Figure 4A). The respective rates using as-observed data were 68.9% (31/45 [95% CI, 55.4–82.4]) and 58.7% (44/75 [95% CI, 47.5–69.8]; Δ = 10.2%; 1-sided *P* = .126), while they were 50.8% (31/61 [95% CI, 38.3–63.4]) in the early and 41.5% (44/106 [95% CI, 32.1–50.9]; Δ = 9.3%; 1-sided *P* = .122) in the delayed cohort, when using the NRI method.

Patients in the early group who were bio-naive had extraintestinal manifestations and steroid-dependent/refractory CD at baseline achieved higher 26-week off-steroids remission rates compared with their counterparts in the delayed group (LOCF imputation; Figure 4B–D).

Using the more strict remission criterion of HBI <4, the 26-week off-steroids remission rates were 55.7% (34/61; [95% CI, 43.3–68.2]) and 37.7% (40/106 [95% CI, 28.5–47.0]; Δ = 18.0%; 1-sided *P* = .011) in the early and delayed cohorts, respectively, using the LOCF imputation method, 66.7% (30/45 [95% CI, 52.9–80.4]) and 49.3% (37/75 [95% CI, 38.0–60.6]; Δ = 17.3%; 1-sided *P* = .028) using as-observed data, and 49.2% (30/61 [95% CI, 36.6–61.7] and 34.9% (37/106 [95% CI, 25.8–44.0]; Δ = 14.3%; 1-sided *P* = .035), respectively, using the NRI method.

The 26-week off-steroids clinical response rate (HBI decrease ≥3) was 68.9% (42/61 [95% CI, 57.2–80.5]) and 62.3% (66/106 [95% CI, 53.0–71.5]; 1-sided *P* = .192) in the early and delayed cohorts, respectively, using LOCF imputation. The rates were 80.0% (36/45 [95% CI, 68.3–91.7]) and 74.7% (56/75 [95% CI, 64.8–84.5]; 1-sided *P* = .247), respectively, using as-observed data, and 59.0% (36/61 [95% CI, 46.7–71.4)] and 52.8% (56/106 [95% CI, 43.3–62.3]; 1-sided *P* = .218), respectively, using the NRI method.

### HBI Scores and CRP Values Throughout the Study

In both the early and the delayed ADL cohorts, the baseline HBI score significantly decreased at all post-baseline timepoints (4-, 12-, 26-, and 52-week post-baseline), considering patients with available paired assessments (see Supplementary Table 1, which shows changes in the HBI score from baseline to post-baseline timepoints among patients with paired baseline and post-baseline assessments). Based on as-observed data, the week 52 off-steroids remission (HBI ≤4) rates were 78.8% (26/33 [95% CI, 64.8–92.7]) and 60.3% (35/58 [95% CI, 47.8–72.9]) in the early and delayed ADL cohorts, and the respective off-steroids clinical response rates were 84.8% (28/33 [95% CI, 72.6–97.1]) and 69.0% (40/58 [95% CI, 57.1–80.9]).

Concerning CRP values, a marginal model with the main effects of time elapsed from ADL onset, ADL cohort, and their interaction, did not reveal a significant interaction between-study cohort and time (*P* = .416). As a result, a marginal model including only the main effects was fitted according to which time elapsed from ADL initiation was statistically significant [specifically, CRP was estimated to decrease by 0.004 (95% CI, −0.008, −0.000; *P* = .026) units per week after the start of ADL treatment], but early vs delayed initiation of ADL treatment was not.

### CD-Related Intestinal Resection and Progression From an Inflammatory to a Stricturing and/or Penetrating Phenotype

During the study look-back observation period, the CD-related intestinal resection rates (for reasons other than perianal complications) were 6.5% (4/62) and 11.9% (13/109) in the early and delayed cohorts, respectively, and the rates of CD progression to a complicated (stricturing and/or penetrating) phenotype were 7.7% (3/39) and 11.4% (8/70). The median Kaplan–Meier estimated time to first intestinal resection was not reached in either cohort (see Supplementary Figure 1A for the Kaplan–Meier curve). The HR of the delayed vs early cohort was 1.81 (95% CI, 0.59–5.56; *P* = .299). The median Kaplan–Meier estimated time to progression to a complicated phenotype was 105.2 months (95% CI, 80.1–105.2) in the early cohort but was not estimable in the delayed cohort (see Supplementary Figure 1B for the Kaplan–Meier curve). The HR of the delayed vs early cohort was 1.33 (95% CI, 0.35–5.09; *P* = .677).

## Discussion

This study has demonstrated that initiation of ADL within the first 2 years following diagnosis in adult patients with moderate to severe CD with at least 3 PPFs resulted in a significantly higher remission rate compared with a delayed ADL introduction.

The effect of disease duration on the efficacy/effectiveness of biologic treatments in patients with CD has been addressed by several studies.^18–22^ However, to the best of our knowledge, this is the first evidence derived from a CD study in a uniform population of White patients with poor prognosis, with another study examining the effect of early anti-TNF introduction in an exclusively Asian population with PPFs.^23^ In addition to the identified demographic, behavioral, disease-specific, comorbid, and genetic prognostic factors, this study involved those indicated by the Annual Exchange on the Advances in IBD (IBD Ahead) 2014 educational program, which included a comprehensive literature review to identify prognostic factors related to long-term outcomes and develop evidence-weighted summary statements.^19^ Smoking,^6, 7, 24^ young age at diagnosis,^2, 7, 25, 26^ ileal disease location,^26–29^ deep and extensive ulceration,^30^ severe rectal and/or perianal disease,^2^ and upper gastrointestinal involvement,^31^ were recognized as predictive for both increased surgery needs and change of CD behavior from inflammatory to stricturing and/or penetrating phenotypes.

Analysis of the study’s primary endpoint of the 26-week remission rates (off-steroids) revealed a statistically significant difference between the early and delayed cohorts. This was more pronounced when using a more stringent definition of remission as the target, indicating the potential of early treatment to achieve even more stringent therapeutic goals. These findings are in line with a subgroup analysis of the CHARM trial, indicating that initiation of ADL within <2 years vs ≥2 years of disease onset offers a significant benefit in remission rates at 26 weeks.^18^ Similarly, in the certolizumab PRECISE 2 trial, the 26-week remission rates were inversely related to CD duration.^32^ Of note, the populations recruited in these randomized clinical trials do not represent a homogenous sampling of patients with poor prognosis, whereas our study does. Similarly to week 6, higher remission rates in the early vs delayed cohort observed were observed at week 52. Such a difference between early and delayed initiation of ADL is also evident in the 56-week remission rates of the CHARM/ADHERE trials of patients with CD regardless of the presence of PPFs at baseline.^18^

Not unexpectedly, in our study, the difference in the remission rates between the early and delayed cohort was more prominent in bio-naive patients. This subpopulation is of special interest because it is exempt from the confounding effect of prior exposure to biologics so that the clinical benefit derived from the earlier initiation of ADL is mirrored closest to its net effect. The greater ADL effect in bio-naive patients is in accordance with that of the SONIC trial, in which the noted benefit of infliximab was at least in part attributed to the inclusion in the study of immunosuppressive and bio-naive patients with a short disease duration (mean of 2.3 years).^33^

Beyond bio-naive patients, significant differences between the early and the delayed cohorts were also observed in subgroups with and without steroid dependency/refractoriness and those with and without extraintestinal manifestations at treatment initiation. From a clinical perspective, these results suggest that early intervention with ADL would be highly beneficial when targeting bio-naive patients and even more beneficial when there is coexistence of steroid dependency/refractoriness and/or extraintestinal manifestations at treatment initiation. In any case, the considerable proportion of patients with extraintestinal manifestations underscores the importance of a multidisciplinary approach towards CD management.

The evaluation of the surgical rates and the rates of progression into complicated phenotypes over time are conceptually associated with the therapeutic mindset of treating promptly within the “window of opportunity” to modify the natural course of the disease, in terms of slowing the progression to stricturing/penetrating phenotypes and/or decreasing the need for CD-related surgeries over time.^34^ In the present study, the rates of CD progression into stricturing and/or penetrating phenotypes as well as the CD-related intestinal resection rates were numerically, but not statistically significantly, lower in the early vs delayed cohorts. Initiation of anti-TNF treatment ≤2 years since CD diagnosis has been previously found to be associated with a reduced risk of developing bowel strictures.^20^ Additionally, in a study of an exclusively Asian population carrying ≥2 PPFs at baseline, a delayed vs early initiation of anti-TNF or immunomodulators was associated with a reduced risk of developing stricturing and penetrating complications.^23^ Further relevant evidence has been generated from a recent retrospective study in which earlier initiation of anti-TNF tended to slow the progression of cumulative bowel damage (*P* = .069), a term not identical, but overlapping with “CD phenotype progression.” ^21^ A greater rate of surgical resection in the late (>2 years of disease duration) compared with the early anti-TNF initiation cohort was reported in a previous retrospective study of similar design.^22^ Similarly, the top-down approach (ie, introduction of anti-TNF agents earlier) has been associated with a lower risk for CD-related surgery.^35^

The 52-week ADL survival rate in our study exceeded 90% in the early and delayed cohorts. This rate is somewhat higher than the 1-year rate of 81% reported in a previous study with ADL^36^ and the 85% rate reported in a study of patients treated with biologic therapy.^37^ The higher rate in our study is likely explained by between-study differences in patient baseline characteristics that affect drug retention rates, including sex, prior use of biologics, disease severity, and the presence of PPF among others.^36^

Main limitations of our study include its retrospective design and the missing rate of paired assessments with regard to the HBI at week 26 (26.2% among the early cohort and 29.2% among the delayed cohort). Nonetheless, the difference in the 26-week remission rates between the 2 cohorts with LOCF, NRI, and as-observed analysis consistently point towards a greater derived benefit from early ADL initiation (higher rates in the early cohort), albeit being statistically significant only when using LOCF analysis. The lack of statistical significance with as-observed data and with NRI is likely due to the relatively high missing rate of observations (26% in the early cohort and 29% in the delayed cohort). Other sources for this variation, such as the reason for missing observations, may also have contributed, but such information is not available to be appraised. Another limitation stems from the use of 1-tailed tests for appraisal of the difference between the 2 cohorts in the HBI remission and clinical response rates, which precludes the possibility of detecting an effect in the opposite direction. Of note, the alternate hypothesis of interest of our study, ie, that early initiation of ADL would offer a greater benefit in 26-week remission rates compared to patients initiated with ADL ≥2 years following diagnosis, which formed the basis of using 1-sided testing, was formulated based on published data available at the time of study planning,^18^ but also based on recommendations for early introduction of anti-TNF therapy in patients with PPFs.^19, 26, 27^ Moreover, results in the present study are primarily based on clinical symptoms, which do not always correlate with disease activity. The study results are also limited by the fact that over the study observation period there was a small number of observed events regarding intestinal surgeries (other than for perianal disease) and progression to a complicated phenotype, resulting in immaturity of the data used in the survival analyses for estimation of the median times to event. Furthermore, the differences between the 2 cohorts in the rates of CD progression into stricturing and/or penetrating phenotypes as well as the CD-related intestinal resection rates did not reach statistical significance, maybe also due to the overall low number of observed events. The low rate of history of CD-related resections prior to ADL initiation, must be appraised taking into consideration the fact that patients with a history of intestinal resection were excluded from enrollment and that minor surgical procedures, such as seton placement, may not have been consistently recorded in patients’ medical charts. It is noted that the main clinical characteristics of the patients did not statistically significantly differ between the 2 cohorts at baseline (Figure 3), thus limiting potential confounding of the study outcomes. It should be acknowledged that 37.1% and 34.9% of patients from the early and delayed ADL cohorts, respectively, already had stricturing/penetrating phenotype at baseline. Although the difference between the 2 cohorts is not statistically significant, these proportions are considerably high, particularly in the early cohort, and may have impacted the results of the study. Furthermore, while the study only examined the impact of early initiation of ADL on disease outcomes in patients with PPFs, the beneficial effects seen in this study may likely be applicable to early initiation of any anti-TNF, which is supported by the more pronounced difference between the 2 cohorts in the bio-naïve populations and is aligned with recommendations for early biologic therapy initiation in patients with CD and PPFs.^19, 26, 27^

The major strength of the study is the use of PPFs when investigating the clinical effect of intervening early in the course of CD. To our perception, prognosis is the tool to individualize the definition of early CD, which can be used as a measure of the length of the patient’s individual “window of opportunity” to intervene and maximize treatment outcomes. We have attempted to use prognosis to tailor a custom-made treatment paradigm for ADL by articulating the patient profile that would benefit most from early ADL initiation. Conceptually, we believe that the clinical implementation of the PPF would protect patients with poor prognosis from being undertreated and patients with favorable prognosis, whose “window of opportunity” may be as long as their lifetime, from being overtreated. However, to gain the most from the clinical implementation of prognosis, the quantitative validation of the prognostic factors should first be determined through relevant studies. Regarding the generalizability of the study results, it is noted that the study population was enrolled from 10 hospital sites located in diverse geographic locations, aiding representativeness and allowing the reflection of variations in medical practice paradigms.

## Conclusion

This real-world, nationwide study emphasizes the short- and medium-term benefits of early vs delayed treatment with ADL in patients with CD who carry a number of PPFs for a long-term disabling disease course. The beneficial effect of early ADL treatment seems to be more pronounced in bio-naive patients for whom treatment has failed or who are dependent on treatment with steroids and experience the devastating effect of severe extraintestinal manifestations. However, well-designed, prospective, real-world studies are needed to confirm our results.

## Supplementary Material

otab064_suppl_Supplementary_Figure_1

otab064_suppl_Supplementary_Table_1

## Data Availability

AbbVie is committed to responsible data sharing regarding the clinical trials we sponsor. This includes access to anonymized, individual- and trial-level data (analysis datasets), as well as other information (eg, protocols and clinical study reports), as long as the trials are not part of an ongoing or planned regulatory submission. This includes requests for clinical trial data for unlicensed products and indications. These clinical trial data can be requested by any qualified researchers who engage in rigorous, independent scientific research, and will be provided following review and approval of a research proposal and Statistical Analysis Plan (SAP) and execution of a Data Sharing Agreement (DSA). Data requests can be submitted at any time and the data will be accessible for 12 months, with possible extensions considered. For more information on the process or to submit a request, visit the following link: https://www.abbvie.com/our-science/clinical-trials/clinical-trials-data-and-information-sharing/data-and-information-sharing-with-qualified-researchers.html.

## Notes

Conference presentation: This work was presented in part at Digestive Disease Week^®^ 2019 (abstract 3148483), San Diego Convention Center, San Diego, CA, USA, May 18–21, 2019.

## References

[1] Cosnes J , Cattan S, Blain A, et al. Long-term evolution of disease behavior of Crohn’s disease. Inflamm Bowel Dis. 2002;8:244–250. 12131607 10.1097/00054725-200207000-00002

[2] Beaugerie L , Seksik P, Nion-Larmurier I, et al. Predictors of Crohn’s disease. Gastroenterology. 2006;130:650–656. 16530505 10.1053/j.gastro.2005.12.019

[3] Dubinsky MC , Lin YC, Dutridge D, et al.; Western Regional Pediatric IBD Research Alliance. Serum immune responses predict rapid disease progression among children with Crohn’s disease: immune responses predict disease progression. Am J Gastroenterol. 2006;101:360–367. 16454844 10.1111/j.1572-0241.2006.00456.x PMC2259248

[4] Cleynen I , González JR, Figueroa C, et al. Genetic factors conferring an increased susceptibility to develop Crohn’s disease also influence disease phenotype: results from the IBDchip European Project. Gut. 2013;62:1556–1565. 23263249 10.1136/gutjnl-2011-300777

[5] Aldhous MC , Drummond HE, Anderson N, et al. Does cigarette smoking influence the phenotype of Crohn’s disease? Analysis using the Montreal classification. Am J Gastroenterol. 2007;102:577–588. 17338736 10.1111/j.1572-0241.2007.01064.x

[6] Sands BE , Arsenault JE, Rosen MJ, et al. Risk of early surgery for Crohn’s disease: implications for early treatment strategies. Am J Gastroenterol. 2003;98:2712–2718. 14687822 10.1111/j.1572-0241.2003.08674.x

[7] Brant SR , Picco MF, Achkar JP, et al. Defining complex contributions of NOD2/CARD15 gene mutations, age at onset, and tobacco use on Crohn’s disease phenotypes. Inflamm Bowel Dis. 2003;9:281–289. 14555911 10.1097/00054725-200309000-00001

[8] Halabi S , Owzar K. The importance of identifying and validating prognostic factors in oncology. Semin Oncol. 2010;37:e9–18. 20494694 10.1053/j.seminoncol.2010.04.001 PMC2929829

[9] Altman DG . Studies investigating prognostic factors: conduct and evaluation. In: Gospodarowicz MK, O’Sullivan B, Sobin H, eds. Prognostic Factors in Cancer. 3rd ed. Wiley-Liss; 2006:39–54.

[10] Ananthakrishnan AN , Binion DG. Editorial: improved efficacy of biological maintenance therapy in “early” compared with “late” Crohn’s disease: strike while the iron is hot with anti-TNF agents? Am J Gastroenterol. 2010;105:1583–1585. 20606661 10.1038/ajg.2010.98

[11] Peyrin-Biroulet L . Why should we define and target early Crohn’s disease. Gastroenterol Hepatol (NY). 2011;7:324–6. PMC3127038 21857834

[12] Peyrin-Biroulet L , Loftus EV Jr, Colombel JF, Sandborn WJ. Early Crohn disease: a proposed definition for use in disease-modification trials. Gut. 2010;59:141–147. 20176633 10.1136/gut.2009.187120

[13] Arnett FC , Edworthy SM, Bloch DA, et al. The American Rheumatism Association 1987 revised criteria for the classification of rheumatoid arthritis. Arthritis Rheum. 1988;31:315–324. 3358796 10.1002/art.1780310302

[14] Aletaha D , Huizinga TW. The use of data from early arthritis clinics for clinical research. Best Pract Res Clin Rheumatol. 2009;23:117–123. 19233051 10.1016/j.berh.2008.11.008

[15] Peyrin-Biroulet L , Panés J, Sandborn WJ, et al. Defining disease severity in inflammatory bowel diseases: current and future directions. Clin Gastroenterol Hepatol. 2016;14:348–354.e17. 26071941 10.1016/j.cgh.2015.06.001

[16] Van Assche G , Dignass A, Panes J, et al.; European Crohn’s and Colitis Organisation (ECCO). The second European evidence-based Consensus on the diagnosis and management of Crohn’s disease: Definitions and diagnosis. J Crohns Colitis. 2010;4:7–27. 21122488 10.1016/j.crohns.2009.12.003

[17] Dignass A , Van Assche G, Lindsay JO, et al.; European Crohn’s and Colitis Organisation (ECCO). The second European evidence-based Consensus on the diagnosis and management of Crohn’s disease: Current management. J Crohns Colitis. 2010;4:28–62. 21122489 10.1016/j.crohns.2009.12.002

[18] Schreiber S , Reinisch W, Colombel JF, et al. Subgroup analysis of the placebo-controlled CHARM trial: increased remission rates through 3 years for adalimumab-treated patients with early Crohn’s disease. J Crohns Colitis. 2013;7:213–221. 22704916 10.1016/j.crohns.2012.05.015

[19] Torres J , Caprioli F, Katsanos KH, et al. Predicting outcomes to optimize disease management in inflammatory bowel diseases. J Crohns Colitis. 2016;10:1385–1394. 27282402 10.1093/ecco-jcc/jjw116 PMC5174730

[20] Safroneeva E , Vavricka SR, Fournier N, et al.; Swiss IBD Cohort Study Group. Impact of the early use of immunomodulators or TNF antagonists on bowel damage and surgery in Crohn’s disease. Aliment Pharmacol Ther. 2015;42:977–989. 26271358 10.1111/apt.13363

[21] Panchal H , Wagner M, Chatterji M, et al. Earlier anti-tumor necrosis factor therapy of Crohn’s disease correlates with slower progression of bowel damage. Dig Dis Sci. 2019;64:3274–3283. 30607690 10.1007/s10620-018-5434-4 PMC7049096

[22] Ma C , Beilman CL, Huang VW, et al. Anti-TNF therapy within 2 years of Crohn’s disease diagnosis improves patient outcomes: a retrospective cohort study. Inflamm Bowel Dis. 2016;22:870–879. 26818419 10.1097/MIB.0000000000000679

[23] Oh EH , Oh K, Han M, et al. Early anti-TNF/immunomodulator therapy is associated with better long-term clinical outcomes in Asian patients with Crohn’s disease with poor prognostic factors. PLoS One. 2017;12:e0177479. 28542298 10.1371/journal.pone.0177479 PMC5441601

[24] Burisch J , Pedersen N, Cukovic-Cavka S, et al.; EpiCom-Group. Environmental factors in a population-based inception cohort of inflammatory bowel disease patients in Europe – an ECCO-EpiCom study. J Crohns Colitis. 2014;8:607–616. 24315795 10.1016/j.crohns.2013.11.021

[25] Romberg-Camps MJ , Dagnelie PC, Kester AD, et al. Influence of phenotype at diagnosis and of other potential prognostic factors on the course of inflammatory bowel disease. Am J Gastroenterol. 2009;104:371–383. 19174787 10.1038/ajg.2008.38

[26] Solberg IC , Vatn MH, Høie O, et al.; IBSEN Study Group. Clinical course in Crohn’s disease: results of a Norwegian population-based ten-year follow-up study. Clin Gastroenterol Hepatol. 2007;5:1430–1438. 18054751 10.1016/j.cgh.2007.09.002

[27] Thia KT , Sandborn WJ, Harmsen WS, et al. Risk factors associated with progression to intestinal complications of Crohn’s disease in a population-based cohort. Gastroenterology. 2010;139:1147–1155. 20637205 10.1053/j.gastro.2010.06.070 PMC2950117

[28] Kiss LS , Papp M, Lovasz BD, et al. High-sensitivity C-reactive protein for identification of disease phenotype, active disease, and clinical relapses in Crohn’s disease: a marker for patient classification? Inflamm Bowel Dis. 2012;18:1647–1654. 22081542 10.1002/ibd.21933

[29] Magro F , Portela F, Lago P, et al.; GEDII. Crohn’s disease in a southern European country: Montreal classification and clinical activity. Inflamm Bowel Dis. 2009;15:1343–1350. 19235885 10.1002/ibd.20901

[30] Allez M , Lemann M, Bonnet J, et al. Long term outcome of patients with active Crohn’s disease exhibiting extensive and deep ulcerations at colonoscopy. Am J Gastroenterol. 2002;97:947–953. 12003431 10.1111/j.1572-0241.2002.05614.x

[31] Lazarev M , Huang C, Bitton A, et al. Relationship between proximal Crohn’s disease location and disease behavior and surgery: a cross-sectional study of the IBD Genetics Consortium. Am J Gastroenterol. 2013;108:106–112. 23229423 10.1038/ajg.2012.389 PMC4059598

[32] Schreiber S , Colombel JF, Bloomfield R, et al.; PRECiSE 2 Study Investigators. Increased response and remission rates in short-duration Crohn’s disease with subcutaneous certolizumab pegol: an analysis of PRECiSE 2 randomized maintenance trial data. Am J Gastroenterol. 2010;105:1574–1582. 20234346 10.1038/ajg.2010.78

[33] Colombel JF , Sandborn WJ, Reinisch W, et al.; SONIC Study Group. Infliximab, azathioprine, or combination therapy for Crohn’s disease. N Engl J Med. 2010;362:1383–1395. 20393175 10.1056/NEJMoa0904492

[34] Pariente B , Cosnes J, Danese S, et al. Development of the Crohn’s disease digestive damage score, the Lémann score. Inflamm Bowel Dis. 2011;17:1415–1422. 21560202 10.1002/ibd.21506 PMC3116198

[35] Rubin DT , Uluscu O, Sederman R. Response to biologic therapy in Crohn’s disease is improved with early treatment: an analysis of health claims data. Inflamm Bowel Dis. 2012;18:2225–2231. 22359399 10.1002/ibd.22925

[36] Tanaka H , Kamata N, Yamada A, et al.; ADJUST Study Group. Long-term retention of adalimumab treatment and associated prognostic factors for 1189 patients with Crohn’s disease. J Gastroenterol Hepatol. 2018;33:1031–1038. 29087616 10.1111/jgh.14034

[37] Jung YS , Han M, Park S, Cheon JH. Biologic use patterns and predictors for non-persistence and switching of biologics in patients with inflammatory bowel disease: a nationwide population-based study. Dig Dis Sci. 2020;65:1436–1444. 31677070 10.1007/s10620-019-05867-1

