# Supplementary material for: The Effect of Early vs Delayed Initiation of Adalimumab on Remission Rates in Patients With Crohn’s Disease With Poor Prognostic Factors: The MODIFY Study
Source: Crohns Colitis 360. 2021 Sep 4;3(4):otab064. doi: 10.1093/crocol/otab064 (PMC9802300; doi:10.1093/crocol/otab064)

Supplementary Figure

**Supplementary Figure 1.** Kaplan-Meier-survival plots in the early and delayed ADL cohorts. (A) Kaplan-Meier survival plot of the intestinal resection–free survival probability. Surgeries due to perianal disease were not included in the analysis. (B) Kaplan-Meier survival plot of Crohn’s disease progression from an inflammatory to a stricturing and/or penetrating phenotype. ADL, adalimumab


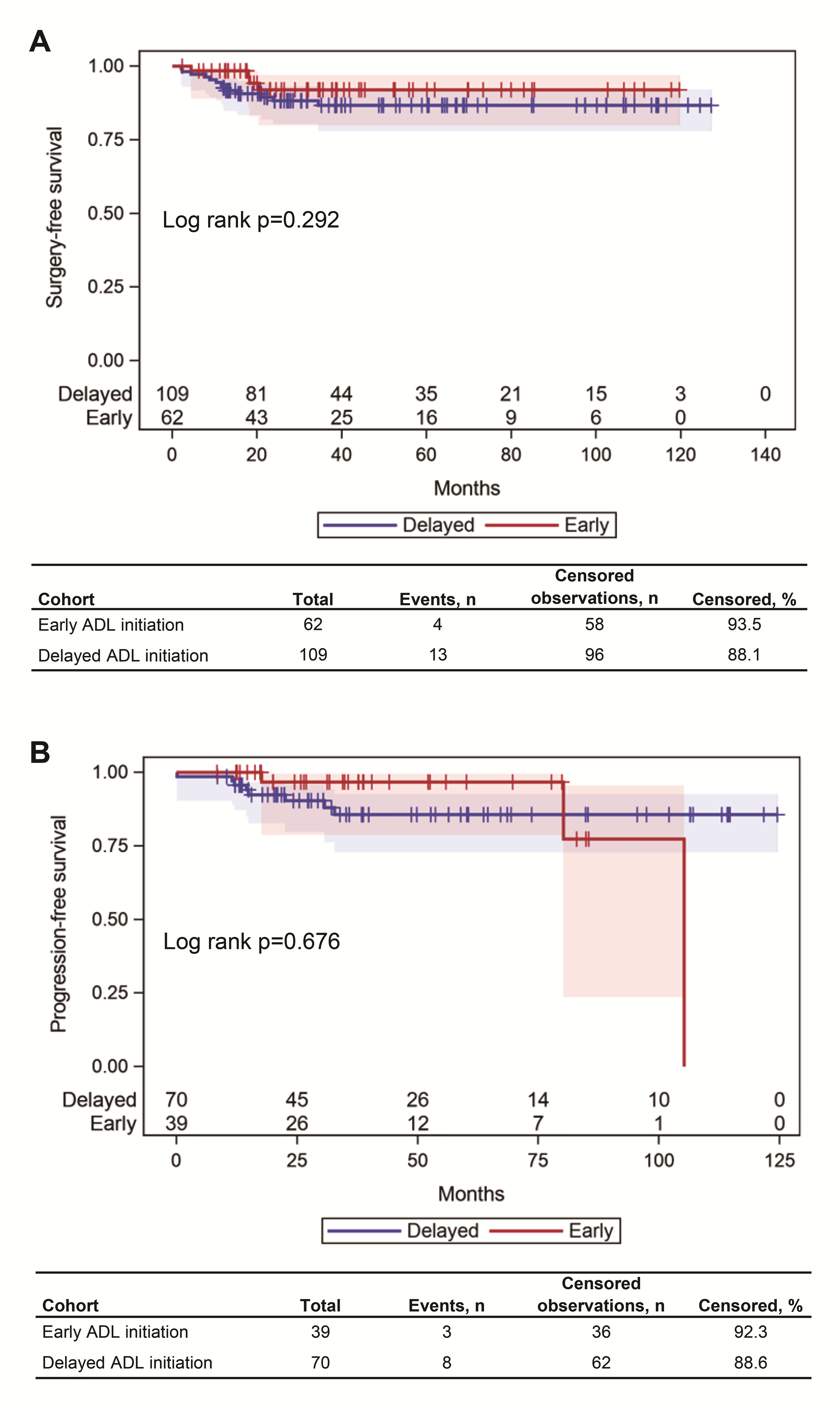

Supplement: otab064_suppl_Supplementary_Figure_1 [file otab064_suppl_supplementary_figure_1.docx]
