# Supplementary material for: The Effect of Early vs Delayed Initiation of Adalimumab on Remission Rates in Patients With Crohn’s Disease With Poor Prognostic Factors: The MODIFY Study
Source: Crohns Colitis 360. 2021 Sep 4;3(4):otab064. doi: 10.1093/crocol/otab064 (PMC9802300; doi:10.1093/crocol/otab064)
Supplement: otab064_suppl_Supplementary_Table_1 [file otab064_suppl_supplementary_table_1.docx]

**Supplementary Table 1.** **HBI score changes between baseline and post-baseline time points in the early and delayed cohorts**

|  | **Early ADL cohort** | | | **Delayed ADL cohort** | | | |
| --- | --- | --- | --- | --- | --- | --- | --- |
|  | **n** | **Median (Q25, Q75)** |  | **n** | **Median (Q25, Q75)** |  | |
| **Baseline HBI score in patients with available data** | 62 | 8.0 (8.0, 9.0) |  | 109 | 9.0 (8.0, 10.0) | |  |
| **Data in patients with paired baseline and post-baseline HBI assessment** | **n** | **Score** | **p value** | **n** | **Score** | **p value** | |
| Paired baseline and Week 4 |  | Mean (SD) |  |  | Mean (SD) | |  |
| Baseline | 29 | 8.8 (1.4) |  | 47 | 9.6 (2.1) | |  |
| Week 4 | 29 | 3.9 (2.9) |  | 47 | 4.5 (2.6) | |  |
| Change from baseline to Week 4 | 29 | –5.0 (3.1) | <0.001 | 47 | –5.2 (2.5) | | <0.001 |
| Paired baseline and Week 12 |  | Median (Q25, Q75) |  |  | Mean (SD) | |  |
| Baseline | 30 | 8.0 (8.0, 9.0) |  | 45 | 9.4 (2.0) | |  |
| Week 12 | 30 | 2.0 (0.0, 3.0) |  | 45 | 3.2 (2.6) | |  |
| Change from baseline to Week 12 | 30 | –7.5 (–8.0, –5.0) | <0.001 | 45 | –6.2 (2.8) | | <0.001 |
| Paired baseline and Week 26 |  | Median (Q25, Q75) |  |  | Median (Q25, Q75) | |  |
| Baseline | 41 | 8.0 (8.0, 9.0) |  | 66 | 8.0 (8.0, 10.0) | |  |
| Week 26 | 41 | 1.0 (0.0, 4.0) |  | 66 | 2.0 (0.0, 5.0) | |  |
| Change from baseline to Week 26 | 41 | –7.0 (–8.0, –5.0) | <0.001 | 66 | –7.0 (–9.0, –5.0) | | <0.001 |
| Paired baseline and Week 52 |  | Mean (SD) |  |  | Median (Q25, Q75) | |  |
| Baseline | 31 | 8.7 (1.4) |  | 51 | 9.0 (8.0, 10.0) | |  |
| Week 52 | 31 | 2.0 (2.6) |  | 51 | 3.0 (1.0, 6.0) | |  |
| Change from baseline to Week 52 | 31 | –6.6 (2.7) | <0.001 | 51 | –7.0 (–9.0, –3.0) | | <0.001 |
| Paired baseline and end of the observation period |  | Mean (SD) |  |  | Median (Q25, Q75) | |  |
| Baseline | 39 | 9.0 (1.5) |  | 56 | 9.0 (8.0, 10.0) | |  |
| End of the observation period | 39 | 3.1 (3.6) |  | 56 | 3.0 (1.0, 6.0) | |  |
| Change from baseline to end of the observation period | 39 | –5.9 (4.0) | <0.001 | 56 | –6.0 (–8.0, –3.0) | | <0.001 |

P values were calculated using *t* test or Wilcoxon signed rank test, as applicable according to the normality of data distribution. Mean (SD) values are presented for data following a normal distribution; median (Q25, Q75) are presented for data not following a normal distribution.

ADL, adalimumab; HBI, Harvey-Bradshaw Index; SD, standard deviation; Q25, 25th percentile; Q75, 75th percentile
